# School Hygiene—II

**Published:** 1880-07

**Authors:** 


The Bistoury.

ELMIRA, N. Y., JULY, 1880.

The BISTOURY is published Quarterly, upon the first of April, July, October and January, at Fifty Cents a year in advance.

### SCHOOL HYGIENE, II.

Much attention has been lavished upon our methods and apparatus for school teaching.—Books, maps and other appliances for the school-room have been wonderfully improved and beautified in recent years, so that it would seem, little remains to be desired in this direction.

With these improvements, however, proper regard has not been paid to the hygienic influences of the school-room. Too little thought has been given to protecting the health of the children. We have been too prone to *push* them in their educational exercises, without noting the effects produced upon their physical developement.

In the last number of the BISTOURY, we undertook to point out the deleterious effects of our educational system upon the eyes of our children, indicating the causes for the alarming increase of myopia and other derangements of vision, and suggested means for preventing these harmful consequences. We desire now to refer briefly to some causes for ill health in children attending the public schools.

We note first, that children are sent to school too young. No child under seven years of age can be compelled to sit still and gaze upon a book for an entire hour without suffering. It is not natural for a child to be quiet. He can not do it if he would, save under severe discipline and fear of punishment. This enforced quietness on the part of the young child, gives rise to ''nervous'' ailments—St. Vitus' dance, epilepsy, and the like. It also endangers him to spinal curvatures and ''spinal irritation.'' If the child is sent to school at all, before he is seven years old, it should be to a private school, where the utmost liberty for play, and frequent out-door intermission is permitted.

Older children suffer mentally and physically from the severe school discipline that requires them to remain quiet in one seat, for an hour or more, their attention closely fixed upon their books, with no permission to move about or communicate with other pupils without receiving marks of disapproval from the teacher and against their good standing in school. This enforced quietness produces ''nervous'' ailments, headaches, ''spinal irritations,'' &c.—We see no remedy for this, other than to have a ten-minutes recess every hour, in which pupils may be permitted to leave their seats, mingle and talk with each other, or engage in calisthenic exercise of some sort. In this manner, the weary muscles can be rested, the congested brain relieved, and the pupil made ready for another hour of study and imprisonment.

There is no reason why our children should not endure the severe school drill to which they are subjected in these days,—''cramming'' and all,—if proper regard is paid to the bodily comfort. This can be accomplished in suitable school buildings—as recommended in the April number—and by frequent intermissions for exercise and rest of body and brain.

